# Potato Wart Isolates from Europe and North America Form Distinct Clusters of Genetic Variation

**DOI:** 10.3390/life13091883

**Published:** 2023-09-08

**Authors:** Ina Röhrs, Marcus Linde, Jaroslaw Przetakiewicz, Avrelia Zelya, George Zelya, Anna Pucher, Hana Tlapák, Thomas Debener

**Affiliations:** 1 Institute for Plant Genetics, Leibniz University Hannover, 30419 Hanover, Germany; menz@genetik.uni-hannover.de (I.R.); linde@genetik.uni-hannover.de (M.L.); 2 Plant Breeding and Acclimatization Institute—National Research Institute, Radzikow, 05-870 Blonie, Poland; j.przetakiewicz@ihar.edu.pl; 3 National Academy of Agrarian Sciences, Institute of Plant Protection, Ukrainian Scientific-Research Plant Quarantine Station, 60321 Boyany, Chernivtskiy Region, Ukraine; 4 Institute for Plant Protection in Crops and Grassland, Julius Kühn-Institute, 14532 Kleinmachnow, Germany; anna.pucher@julius-kuehn.de; 5 Robert Koch-Institute, 13353 Berlin, Germany; tlapakh@rki.de

**Keywords:** genetic diversity, pathogen evolution, molecular marker, pathogen, gene flow, chitrid

## Abstract

We have extended previously published sets of simple sequence repeat markers for *Synchytrium endobioticum,* selected to be polymorphic for the German-standard isolates of pathotypes P1, P2, P6, P8, and P18. These markers also complement the extensive published information on DNA polymorphisms for the mitogenomes of *Synchytrium endobioticum*. This extended set of 35 markers representing 73 alleles differentiated 51 isolates from Europe and North America into three large, well-separated clusters and subclusters using dendrogram analysis, principal coordinates analysis (PCoA), and population substructure analysis using STRUCTURE 2.3.4 software. This suggests a limited number of introgressions of the wart disease pathogen into current potato growing areas, followed by recombination and admixture of populations through human activities. The new markers extend the published marker sets and are useful tools for future analyses of population structure and dynamics in *Synchytrium endobioticum*, which are necessary to understand the biology of the interaction between the pathogen and its potato host and to develop future control strategies.

## 1. Introduction

Potato wart disease is caused by *Synchytrium endobioticum*, which is an obligate biotrophic chytrid fungus that infects below-ground tubers. Infected tubers develop a tumor-like growth on the emerging sprouts, causing significant yield losses [1,2,3]. As a below-ground disease, potato wart is difficult to control with conventional plant protection measures. Potato wart is classified as a quarantine disease due to its extremely resistant dormant spores, which remain virulent in the soil for several decades [1,4]. In addition to implementing strict phytosanitary measures, breeding resistant potato varieties is a viable strategy to reduce the spread of the fungus in potato growing areas [1].

After the disease was first described in Europe and North America, potato varieties resistant to the first identified pathotypes were developed [1,3]. However, a number of new pathotypes have emerged since then, and only a small number of cultivated potato genotypes carry effective resistance genes against most of the pathotypes [5]. 

As the virulence patterns of pathogens are mostly determined by specific factors called avirulence genes, molecular markers that are not linked to these factors are suitable for studies of genetic relatedness but cannot predict pathotypes [6,7]. Nevertheless, information on the genetic diversity of plant pathogens is very useful for analyzing the geographical differentiation of pathogen populations [8,9]. In addition, information on significant differences in the genetic diversity of *S. endobioticum* populations could not only provide a deeper understanding of the biology of the pathogen, but could also help to understand the dynamics of the evolution of new pathotypes and the spread of genotypes, mainly due to human activities.

The first molecular analyses of *S. endobioticum* aimed at the specific and sensitive detection of the pathogen in infected plant tissues and soil samples [10,11,12,13,14]. In addition, a number of molecular markers have been developed to identify genotypes and characterize the genetic diversity of groups of genotypes of different sizes [15,16]. To date, two complete genomes and partial genomic and transcriptomic datasets have been published for *S. endobioticum,* with assembly sizes of 21.48 and 23.21 Mb [17]. Along with the published nuclear genomes, the structure of the mitochondrial genome, a most likely maternally inherited linear molecule of 72.9 Kbp, has been published, and comparative analyses with the mt genomes of a number of genotypes have been performed [18,19]. These studies showed considerable variation between mitogenomes, with six different haplotypes identified, as well as putative admixtures in some of the populations studied. Furthermore, these authors concluded that there have been at least three introductions of *S. endobioticum* into Europe, and that pathotypes 2(G1) and 6(O1) probably emerged twice independently. The center of origin of *S. endobioticum* is probably the Andean region. The six Peruvian samples analyzed by van de Vossenberg et al. [19] showed mitogenomic sequences not detected in any of the other 136 mitogenomes.

The goals of our present study were:

- To develop and test an extended set of nuclear SSR markers for the analysis of genetic diversity in *Synchytrium endobioticum;*
- To analyze genetic differentiation in a set of 51 isolates from diverse geographic origins, mostly from Europe, with high reliability using a combination of the new and published markers and various computational methods;
- To search for potential diagnostic markers characteristic of the distinct clusters, useful for the characterization of new undefined field samples.

## 2. Materials and Methods

### 2.1. Potato Wart Isolates

A total of 51 samples originating from different geographical regions and representing different pathotypes were analyzed in this study (Table S1). Six isolates with the pathotypes 1(D1), 2(G1), 6(O1), 8(F1),18(T1), and 38(Nevsehir) were obtained from the JKI collection in Kleinmachnow (Germany), representing the German pathotypes P1, P2, P6, P8, and P18 [15], and the “higher” pathotype 38, originating from Turkey. DNA samples from a further 45 isolates were obtained from the IHAR isolate collection (Poland). The three Canadian isolates, 16 to 18, with pathotypes 6 and 8, were originally derived from the ACIA [20,21]. In three of the samples, which were isolated from soil samples, a mixture of two different pathotypes was detected by the IHAR. Sample 24----1(D1)&13(R2)_#UA/5/2020 was a mixture of pathotypes 1(D1) and 13(R2), whereas the soil sample 35----3(M1)&1(D1)_#PL/1/2020 was a mixture of pathotypes 3(M1) and 1(D1). The third mixed sample was 50----1(D1)&38(Nevsehir)#GE/3/2021 with higher pathotypes 38(Nevsehir) and 1(D1). The DNA was isolated from 100 mg aliquots of fresh warts, which were frozen in liquid nitrogen and homogenized with a bead mill, using a DNeasy Plant Mini Kit (Qiagen, Hilden, Germany) according to Busse et al. [15].

### 2.2. Simple Sequence Repeat Markers

Primer design for the new SSRs was performed using Primer 3 software (https://bioinfo.ut.ee/primer3-0.4.0/primer3/) (accessed on 19 November 2020) on the genomic P18 contigs (accessions PRJNA280048, PRJNA453734 and PRJNA453731 at NCBI) as previously described [15]. In addition to the new SSRs, 21 previously published SSRs [16] were included in this study. SSR-PCR was performed using M13 tags at the 5’-end of the locus-specific primers and in a two-step PCR reaction with infrared-labeled M13 primers. PCR products were analyzed via polyacrylamide gel electrophoresis on LI-COR automated sequencers according to Busse et al. [15].

### 2.3. Data Analysis

Marker alleles were transferred to a matrix of 1 (present) or 0 (absent) in MS Excel (Microsoft) and exported to text files compatible with the requirements of the downstream analysis software. In the few cases where isolates showed a second DNA fragment, the prominent fragment was used for scoring, and the minor fragment was omitted. In the case of two fragments of equal intensity, the data point was scored as missing data. Dendrograms were constructed with Darwin 6.0.21 [22] using three different distance measures (Dice, Jaccard, and Sokal & Sneath (un2)) and two different tree construction algorithms (weighted neighbor-joining and UPGMA). Edge lengths were re-estimated so that the distances in the trees were least-squares estimators of the initial dissimilarities. The tree topologies were tested using bootstrap analysis with 1000 replicates. The dendrogram was plotted using Interactive Tree Of Life (iTOL) version 6.7.6 (https://itol.embl.de) (accessed on 6 July 2023) [23] or using Darwin 6.0.21 [22]. PCoA plots were calculated with Darwin 6.0.21 using the Dice dissimilarity index [22] and plotted in 3D using the online tool cubemaker (http://alexpreynolds.github.io/cubemaker) (accessed on 6 July 2023). The population structure was analyzed using Structure 2.3.4 [24] with a burn-in period of 100,000 or 200,000 MCMC replicates after burn-in, with *K* values ranging from 2 to 7, and 30 replicates per *K* value. The results were further analyzed using Structure Harvester via a web application (https://taylor0.biology.ucla.edu/structureHarvester/) (accessed on 13 June 2023).

## 3. Results

### 3.1. Development of New SSR and SCAR Markers

In a previous study, we developed five SSR markers and one SCAR marker that were polymorphic among five German isolates representing pathotypes P1, P2, P6, P8, and P18 [15]. These markers were developed from the partial genome sequences of two pathotype 18 isolates. As a total of 200 SSRs were identified in Busse et al. [15], we now screened the remaining 159 additional SSR motifs for reproducible PCR patterns in a small set of isolates (pathotypes P1, P2, P6, P8, and P18). Of these, 28 primer combinations produced single PCR fragments and showed polymorphisms among the five German isolates (Table S1 and S2). Two additional SCAR markers were developed from an amplicon generated from the published AvrSen1 gene [25] and from SNPs detected between the P18 [15] and MB42 [17] sequence contigs (Table S2). We also screened 21 markers published in a previous study of Canadian and European wart isolates [16] for polymorphisms in our test set of German isolates. We found 11 SSRs with both clear and reproducible amplicons and polymorphisms in the test set of five German isolates (Table S1).

Using a total of 35 markers (5 from Busse et al. [15], 11 from Gagnon et al. [16], and 19 from this study) representing 73 marker alleles, we analyzed a set of 51 wart isolates using PCR and the subsequent separation of amplicons on LI-COR automated sequencers. These isolates were derived from different geographical regions, mostly in Europe, and represented at least ten different pathotypes.

**Figure 1:**
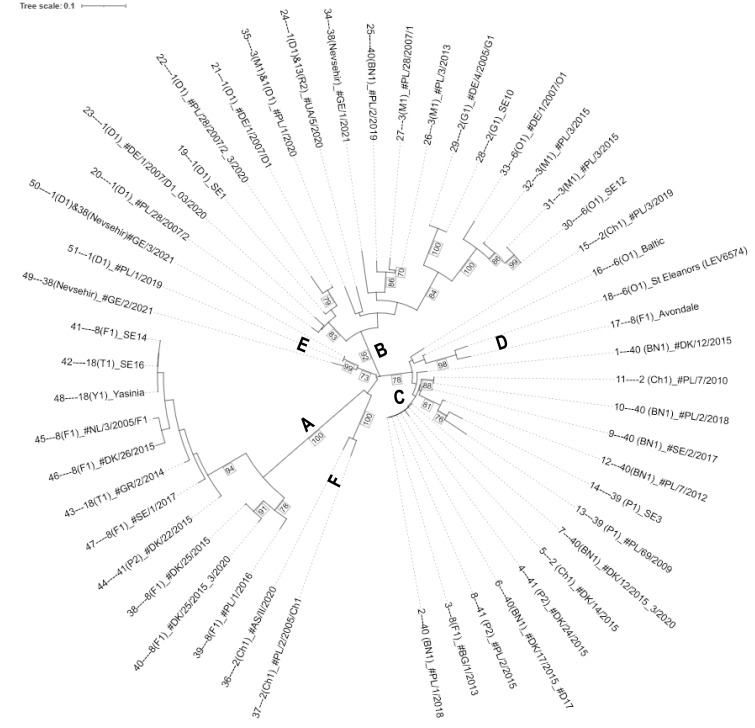
Weighted neighbor-joining dendrogram based on the Dice dissimilarity index for the 51 isolates using 73 marker alleles. Edge lengths are least-squares estimates of the initial dissimilarities. A bootstrap analysis was performed with 1000 replicates, and values above 70% are shown at the branches. The labels A to F indicate six distinct clusters supported by high bootstrap values.

Based on the combined information from all 73 markers (Table S3), we could reproducibly separate all but five groups of isolates (three pairs of isolates, one group of three isolates, and one group of six isolates), which had identical marker patterns. For the SSR marker SSR_54_4845, we found two alleles each for the isolates 45__8(F1)NL/3/2005/F1 and 39__8(F1)DK/26/2015, which showed only weak amplification. As both isolates were sampled in two different countries 10 years apart and are not extremely similar genetically (see Figure 1), we treated these cases as evidence of an admixture of isolates and discarded the weaker band from further analysis. In another case (SSR marker SSR_109_79482), we found two equally intense fragments in isolate 11 (M1), suggesting either diploid material (although the DNA was isolated from warts and not from diploid resting spores) or admixture, so it was excluded from further analysis.

### 3.2. Analysis of Genetic Diversity of Wart Isolates

Neighbor-joining and UPGMA dendrograms were constructed using distance values generated by the Dice, Jaccard, and Sokal & Sneath (un2) indices (Figure 1 and Figure S1). The topology of the neighbor-joining tree based on the Dice dissimilarity index comprised a number of clusters with nodes supported by high bootstrap values, among which three larger highly distinct clusters (A, B, and C) and several subclusters could be identified (Figure 1).

The most distinct group, in terms of high bootstrap value and distance from other groups, consisted of ten isolates of pathotypes 8 and 18 (Figure 1, cluster A). Here, only one isolate (44__41(P2)#DK/22/2015) did not belong to either pathotype 8 or 18. The small cluster F, with two isolates of pathotype 2 separates at a basal node from cluster A. Cluster E contains one isolate with pathotype 1(D1), the isolate 49---38(Nevsehir)_#GE/2/2021, and the soil sample 50---1(D1)&38(Nevsehir)#GE/3/2021 with a mixture of both pathotypes 1 and 38.

Another more heterogeneous cluster with 17 isolates is group B, which contains mainly pathotypes 1, 2, 3, and 6 and a single isolate each of pathotypes 38 and 40. Within this group, a well-supported subcluster was identified with five isolates of pathotype 1 (D1). Interestingly the two soil samples 24----1(D1)&13(R2)_#UA/5/2020 and 35----3(M1)&1(D1)_#PL/1/2020 with a mixture of pathotypes form a separate, but not highly supported, subcluster. 

A third group is formed by clusters C and D, which contain a mixture of isolates, including isolates with higher pathotype numbers. It is noteworthy that seven isolates in cluster C, representing different pathotypes, are either genetically identical or very similar according to our marker data, although they come from different geographical origins and belong to different pathotypes. The subcluster D consists of two Canadian isolates representing pathotypes 6 and 8.

Additional dendrograms constructed using slightly different methods based on different distance measures (Jaccard and Sokal & Sneath coefficients) and/or a different dendrogram generation algorithm (UPGMA) showed essentially the same clusters A, B, and C, with only minor differences in the arrangement of the three other subclusters relative to the first three clusters (Figure S1) and in branch lengths.

**Figure 2:**
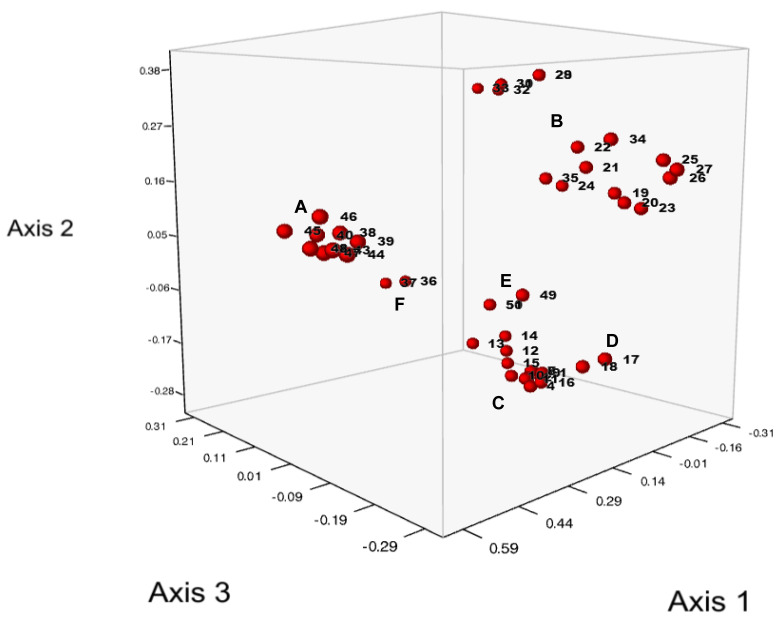
Principal coordinate analysis using Jaccard distances showing the first three axes. The labels A to F mark the six distinct clusters shown in the weighted neighbor-joining dendrogram in Figure 1.

A principal coordinate analysis (PCoA) of the dataset (Figure 2) clearly separates groups of isolates corresponding to clusters A to F from the weighted neighbor-joining dendrogram using Dice dissimilarity. Subcluster D, with the two Canadian isolates of pathotypes 6 and 8, is not as clearly separated from cluster C as in the dendrogram. The first three PCoA axes explained 73.54% of the cumulative variance, with 42.28%, 22.92% and 8.34% of the genetic variation, for the first, second, and third axes, respectively.

**Figure 3:**
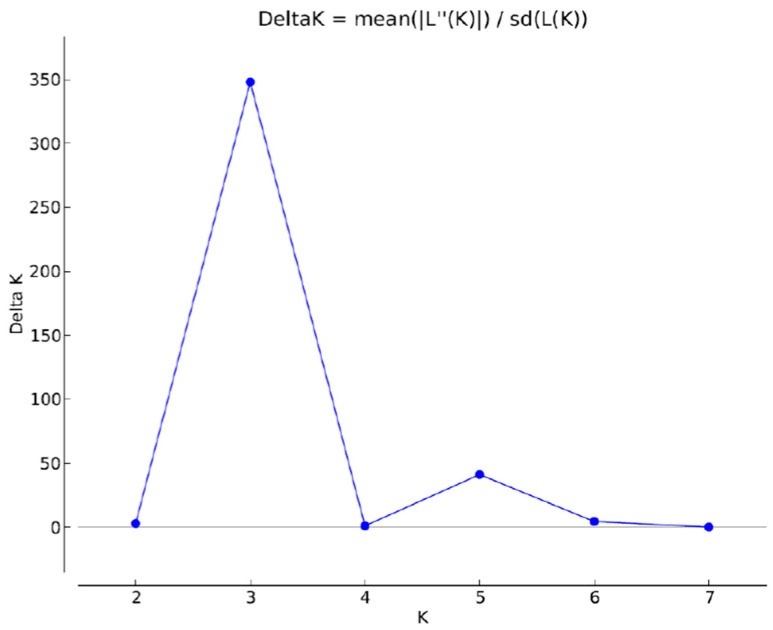
Estimation of the number of subpopulations using the method of Evanno et al. [26] with 1 *K* (*y*-axis) as a function of *K* (mean ± SD) (*x*-axis) using Structure Harvester. The peak at *K* = 3 indicates three subpopulations as the most likely scenario.

**Figure 4:**
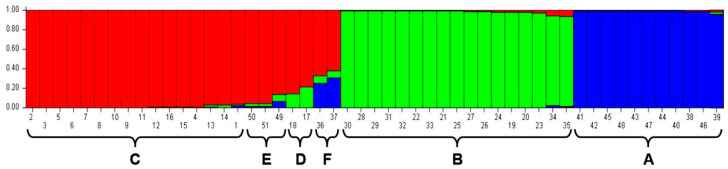
Population structure showing the three identified main clusters A, B, and C and the three additional smaller subclusters D to F for the 51 wart isolates analyzed with 73 marker alleles plotted for K = 3 using STRUCTURE 2.3.4. Each color represents a different subpopulation.

The STRUCTURE analysis also revealed three main groups/subpopulations supported by the dK method (Figure 3 and Figure 4; Evanno et al. [26]).

These correspond perfectly with the clusters defined by PCoA and with groups A, B, and C in the dendrograms. While there is no evidence of admixture for subpopulation A in the STRUCTURE analysis, isolates 34__38 (Nevsehir)#_Ge/1/2021 and 35----3 (M1)_#PL/1/2020 in STRUCTURE subpopulation B show minor genomic admixtures of subpopulations A and C. The STRUCTURE subpopulation C groups the same isolates as in the PCoA and in the dendrograms. Here, isolates forming the distinct subclusters D and F in the dendrograms show evidence of greater admixture from STRUCTURE subpopulations B or A, respectively, whereas subcluster E, with three isolates, shows only smaller admixtures (Figure 4).

### 3.3. Markers Specific to Subclusters A and B

Of the 35 markers, 10 markers showed distinct patterns with alleles specific for particular subclusters. In particular, seven markers (SSR 16161, SSR 818598, SSR 4865, SSR 11176, SSR 122283, SE_29, and SCAR_E271) showed specific alleles for subcluster A, supporting the distinct genetic structure of this subcluster from the other isolates. Within subcluster A, two markers (SSR 7 and SE_04) show fragments specific to the larger subcluster of the isolates 41 to 48. Within cluster B, marker SE_44 is specific for a distinct subset of pathotypes 3 and 6 within this subcluster.

## 4. Discussion

In this study, we extended our previous set of SSR markers by 19 new markers and markers published by others. We achieved a high reliability of diversity measures, as judged by high bootstrap values for the major clusters of the dendrograms and by agreement of the major clusters between the dendrograms, and PCoA and STRUCTURE analysis. This is comparable in resolution to the study by Gagnon et al. [16] in which the 21 SSR markers identified highly supported clusters among 22 *S. endobioticum* isolates [15,16], except that we analyzed more than twice the number of isolates.

Using different clustering methods, PCoA and STRUCTURE analysis, we were able to show that the set of 51 isolates can be reproducibly divided into three main clusters, each of which is subdivided into further subclusters. 

Cluster A represents only isolates of pathotypes 8 and 18, with the exception of one isolate of pathotype 41 (44_41 (P2)_#DK/22/2015). A recent publication analyzing the genetic diversity of potato wart mitogenomes [19] also reports a specific haplotype cluster found only in pathotypes 8 and 18. The clear separation from the other clusters (Figure 1 and Figure 2) suggests a common origin of these isolates. As mitogenomes are most likely maternally inherited, our study complements and confirms these results with data from genomic markers and suggests that the genetic differentiation of pathotypes 8 and 18 from other isolates extends to both the nuclear and the mitogenome. Furthermore, previous analyses of pathotypes 8 and 18 using nuclear markers included only two such isolates, whereas our analysis comprised ten isolates, further supporting the genetic differentiation of these pathotypes. This strengthens previous suggestions of an independent introduction of the two pathotypes 8 and 18 rather than their emergence through de novo mutations [19]. The position of one isolate of pathotype 41 in cluster A could either be the result of a de novo mutation of the avirulence functions that characterize the P8/P18 isolates of this cluster or the result of repeated mating after pathotype 41 was brought into contact with P8/P18 isolates. In the latter case, a simple first-generation hybrid can be ruled out as, otherwise, this isolate would be close to but outside cluster A. This is supported by both the PCoA and the structure plot, where this pathotype 41 isolate is located between the other cluster A isolates (Figure 1 and Figure 2) and shows no evidence of other genome components (Figure 4). However, it cannot be excluded that individual cases of contamination between isolates may have occurred during the process of propagation of isolates on potato varieties, which generally lack resistance genes to most isolates. Other exceptions are two pathotype 8 isolates, one in cluster C (isolate 3---8(F1)_#BG1/2013) and another outside cluster C in cluster D (the Canadian isolate 17---8(F1) Avondale). 

Cluster B contains isolates from P1, P2, P3, and P6, and one isolate each from P38 (34---38(Nevsehir)_#GE/1/2021) and P40 (25----40(BN1)_#PL/2/2019). With the exception of the latter two isolates, no isolates of higher pathotypes were found in this cluster. Here, as in cluster A, groups of isolates most likely represent a common origin, and the two exceptions observed can be explained by recombination after movement of isolates by humans. Explaining the occurrence of a P38 isolate in cluster B via a de novo mutation of another isolate of cluster B seems unlikely as it is one of two isolates of P38 (isolates 34 in cluster B and 49 in cluster E), both sampled at the same time in Georgia and Turkey. As both were sampled from winter sporangia but grouped in different clusters, they could represent segregants after mating with other pathotypes. This is supported by the results of van der Vossenberg et al. [19], who speculated that pathotype 38 (Nevşehir) arose from a mixture of P18(T1) with P2(G1) or P17(M2) by sharing the rare mitogenome δ variant with pathotype 2(G1) and the β variant with pathotype18(T1). However, in cluster B, we found pathotype 38 (Nevşehir), which was clearly separated with high bootstrap support from the pathotype 2(G1) subcluster. In contrast to van der Vossenberg et al. [19], the two pathotype 18(T1) isolates, 42__18(T1)_SE16 from Germany and 43__18(T1)_#GR/2/2014 from Greece, are located in our cluster A.

Cluster C, within which cluster D is separated by high bootstrap values in all dendrograms (Figure 1 and Figure S1), also forms a third subpopulation in the PCoA and structure analysis. Here, a number of isolates representing different pathotypes (P2, P8, P40, and P41) in cluster C are indistinguishable from our marker set. This can be interpreted in two ways: One reason could be that the DNA does not match the pathotype designation because it is derived from warts of potato genotypes that allow the growth of different pathotypes and the DNA is from an admixture of pathotypes in these warts. Another reason may be that some higher number pathotypes (e.g., P40 and P41) are derived from lower number pathotypes (e.g., P8 and P2) by single gene mutations in the corresponding Avr genes. As there is little evidence of admixture in the structure plots, recombination events following the hybridization of different pathotypes seem unlikely.

Knowledge of the genetic structure of a pathogenic species is important for understanding the origin of populations, phylogenetic relationships, and the evolutionary potential of a pathogen [27,28,29]. It can be assumed that the potential to evolve new pathotypes increases, with both the potential to recombine in sexual progeny and its spatial mobility [27]. As *S. endobioticum* has a soil-borne life cycle, mobility is dependent on agronomic practices and can be influenced by sanitation strategies [30]. The three main groups of isolates identified in our study show signs of significant gene flow, as each cluster comprises isolates from geographically distinct regions. This is most likely the result of human activities, such as the transport of infected tubers or soil, and has been reported several times [31,32]. Signs of recombination could be seen in cases such as pathotype 2, where clusters of isolates of the same pathotype are clearly separated, and in the case of subclusters D and F (Figure 4), where there are some signs of genomic admixture, indicating recombination. As only single marker fragments were detected in these isolates, mixtures of DNA from different isolates can be excluded as an explanation.

It is noteworthy that in contrast to previous reports, which explained frequent polymorphisms within isolates as the diploid nature of winter sporangia [16], we only detected two alleles per sample in two exceptions. As our samples were almost entirely derived from DNA isolated from fresh warts, this could be partly explained by the fact that the fungus produces mostly haploid tissue during the development of summer sori as opposed to the diploid winter sporangia. However, this would only be expected if infection events by single zoospores led to the formation of single warts. Another explanation would be that studies based on resting spore populations sampled mixtures of isolates present at infected sites or in complex compost samples. Interestingly, soil samples with known mixes of the pathotype 1(D1) and the two different pathotypes 13(R2) or 3(M1) were grouped in a separate cluster (24----1(D1)&13(R2)_#UA/5/2020 and 35----3(M1)&1(D1)_#PL/1/2020) in cluster B or as for the soil sample 50----1(D1)&38(Nevsehir)#GE/3/2021 together with isolates of the pathotype 1(D1) and 38(Nevsehir) in subcluster E. Therefore, a suspected mixture of pathotypes in samples could at least be supported by the molecular data using the markers presented here. If the maternal inheritance of the mitogenomes can be confirmed in future studies, the combined information from polymorphisms derived from mitogenomes and nuclear markers may also help to distinguish admixtures of isolates from the presence of diploid stages of the fungus, indicating recombination events between genetically distinct genotypes. This information would be crucial for analyzing the adaptive potential of the pathogen.

The separation of most isolates of pathotypes 8 and 18 into subcluster A of our dendrograms is accompanied by seven cluster-specific markers. These markers have the potential to characterize field isolates of P8 and P18. However, the isolates that do not follow the cluster-specific marker patterns need further scrutiny before definitive statements about the specificity of the markers can be made.

## 5. Conclusions

Our extended set of 35 molecular markers grouped DNA samples from 51 *Synchytrium endobioticum* isolates into three main clusters and at least three further subclusters with significant statistical support. This expands the range of nuclear markers available for the genetic characterization of potato wart isolates. A comparison of three different methods all supports two well-defined and one broader subcluster C with a few genotypes showing genomic admixture. As these isolates originate from a wide range of European countries and North America, mixed clusters are most likely the result of gene flow via trade activities between countries. Clearly separated clusters, such as the well-separated main clusters A and B, suggest a common genetic origin. Future analyses should include a larger set of isolates from other geographical regions and more information on polymorphisms derived from characterized Avr genes in order to better understand the population dynamics of potato wart disease and the potential for the evolution of new virulent pathotypes.

## Data Availability

All scoring data of the SSR patterns needed for the genetic diversity analyses are provided in Table S3: Scoring pattern of the 73 markers.

## Supplementary Materials

The following supporting information can be downloaded at: https://www.mdpi.com/article/10.3390/life13091883/s1, Figure S1: Additional dendrograms constructed using Jaccard and Sokal & Sneath coefficients as distance measures and weighted neighbor joining and UPGMA as dendrogram generation algorithms; Table S1: List of genotypes; Table S2: Primer sequences; Table S3: Scoring pattern of the 73 markers.
