# Supplementary material for: Potato Wart Isolates from Europe and North America Form Distinct Clusters of Genetic Variation
Source: Life (Basel). 2023 Sep 8;13(9):1883. doi: 10.3390/life13091883 (PMC10532758; doi:10.3390/life13091883)
Supplement: Supplementary file 1 [file life-13-01883-s001.zip › Figure S1 Additional dendrograms.pdf]

Figure S1: Additional dendrograms constructed using Jaccard and Sokal & Sneath coefficients as distance measures and weighted neighbor joining and UPGMA as dendrogram generation algorithms.

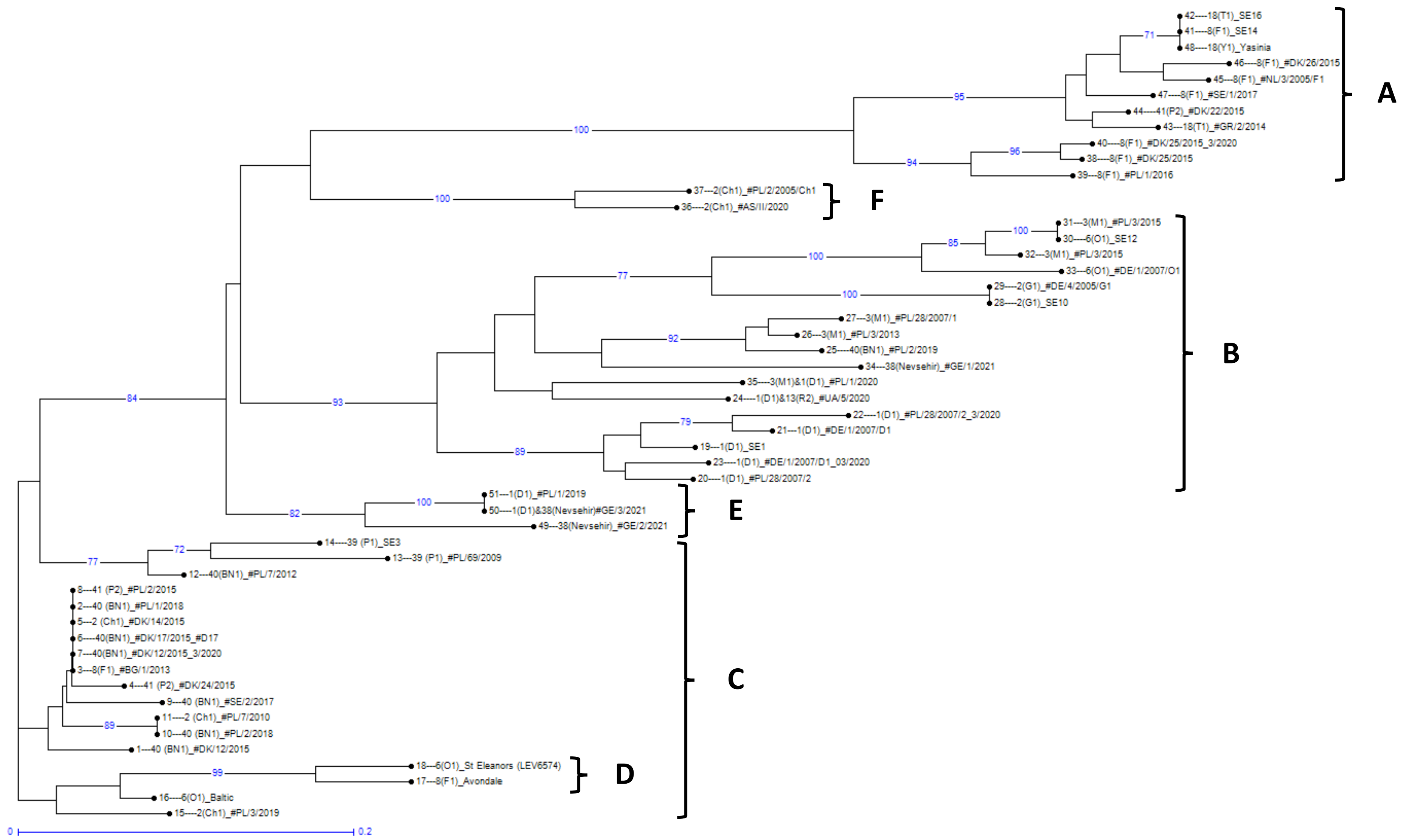

Figure S1a: Weighted Neighbor-Joining dendrogram based on the Jaccard dissimilarity index for the 51 isolates using 73 marker-alleles. A bootstrap analysis with 1000 replicates was applied and values above 70% are shown at the branches.

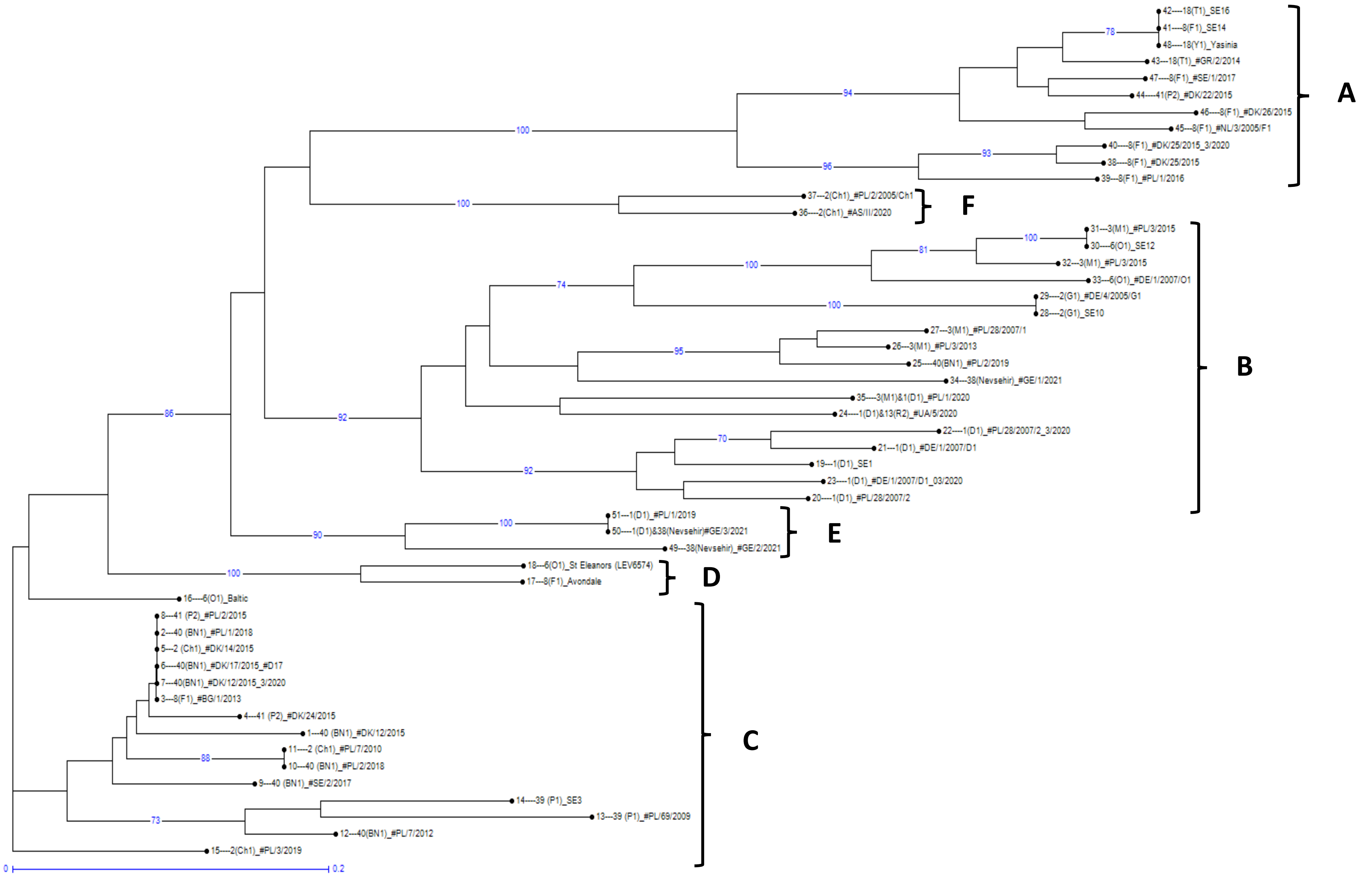

Figure S1b: Weighted Neighbor-Joining dendrogram based on the Sokal & Sneath dissimilarity index for the 51 isolates using 73 marker-alleles. A bootstrap analysis with 1000 replicates was applied and values above 70% are shown at the branches.

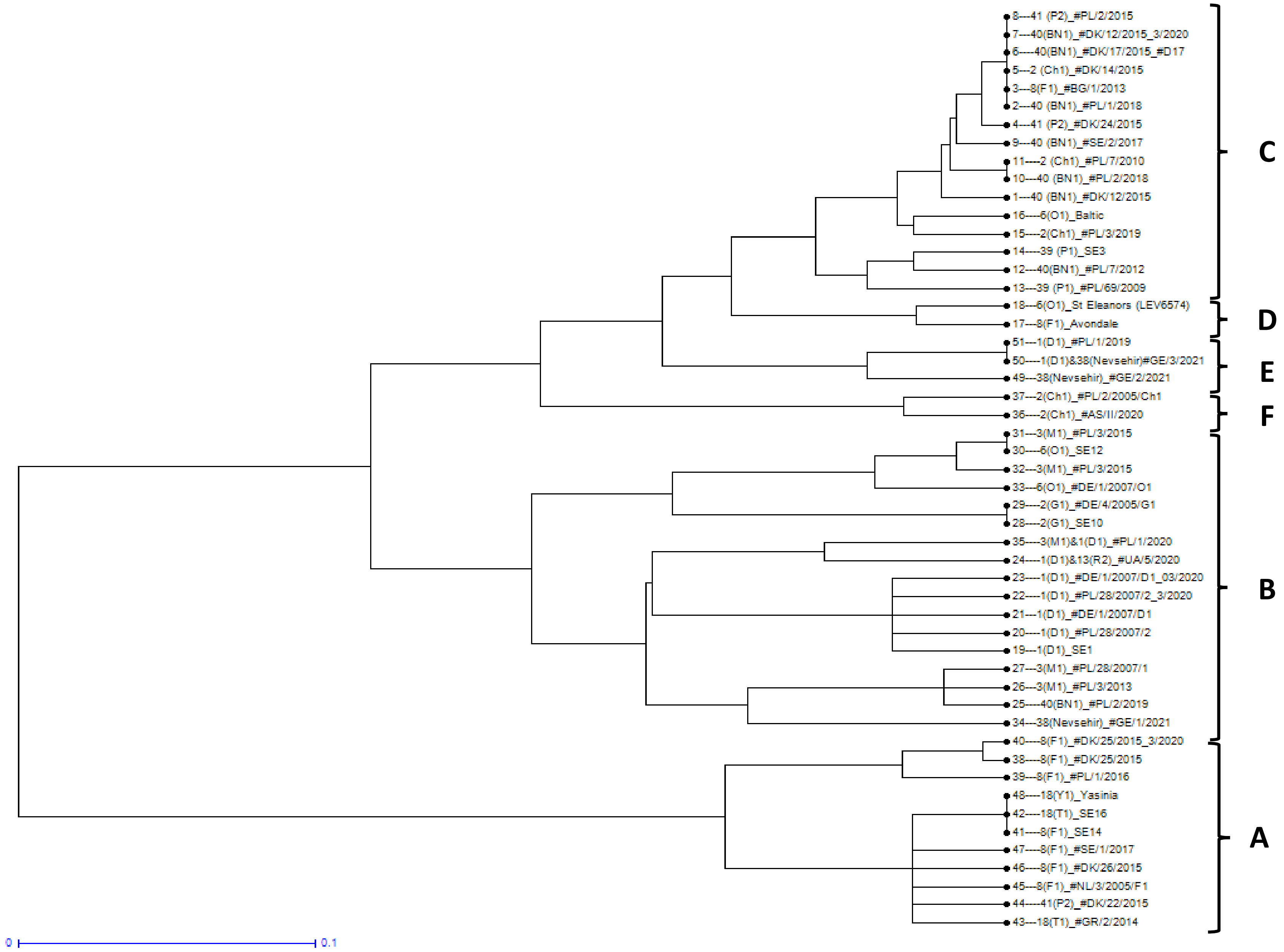

Figure S1c: UPGMA dendrogram based on the Dice dissimilarity index for the 51 isolates using 73 marker-alleles.

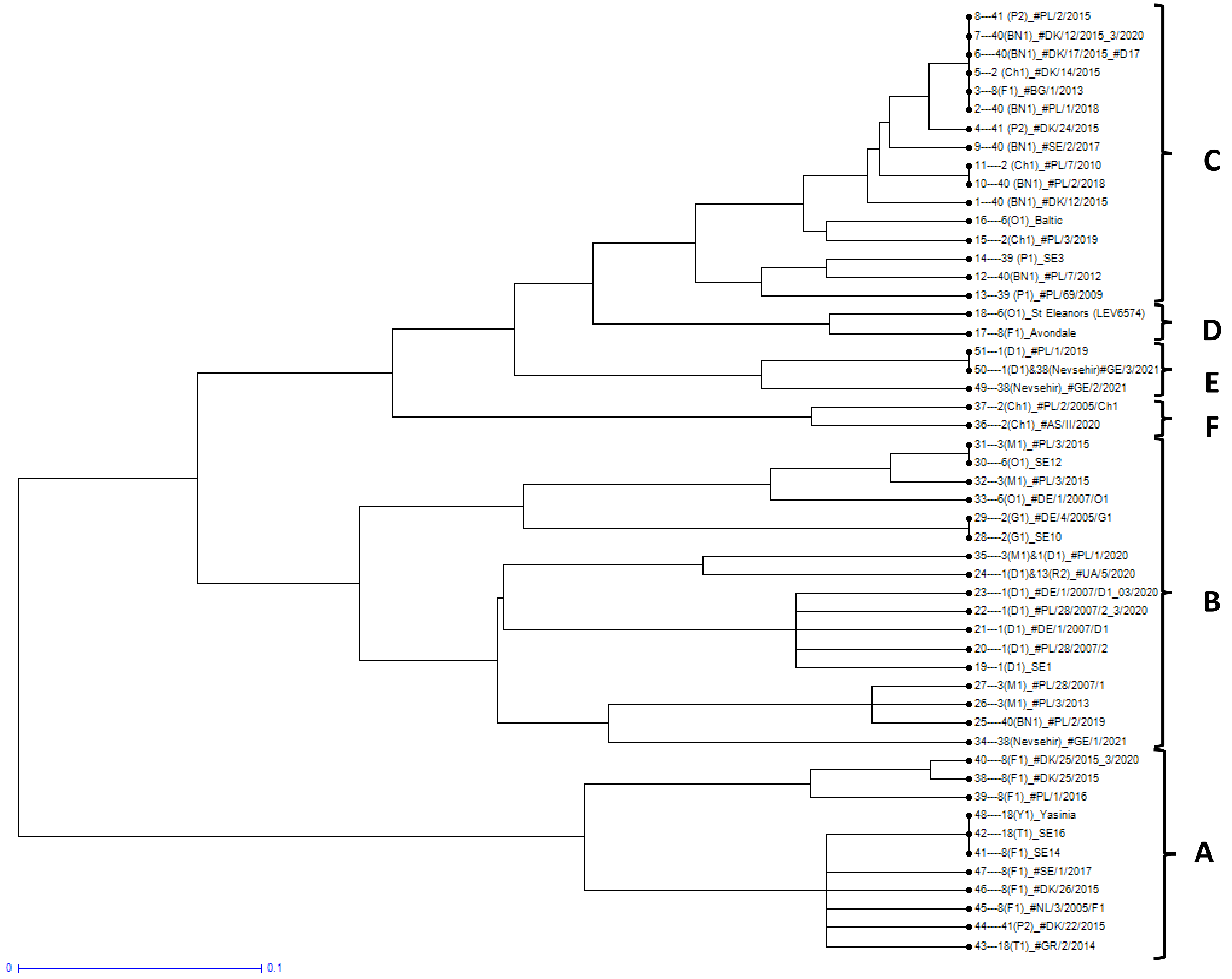

Figure S1d: UPGMA dendrogram based on the Jaccard dissimilarity index for the 51 isolates using 73 marker-alleles.

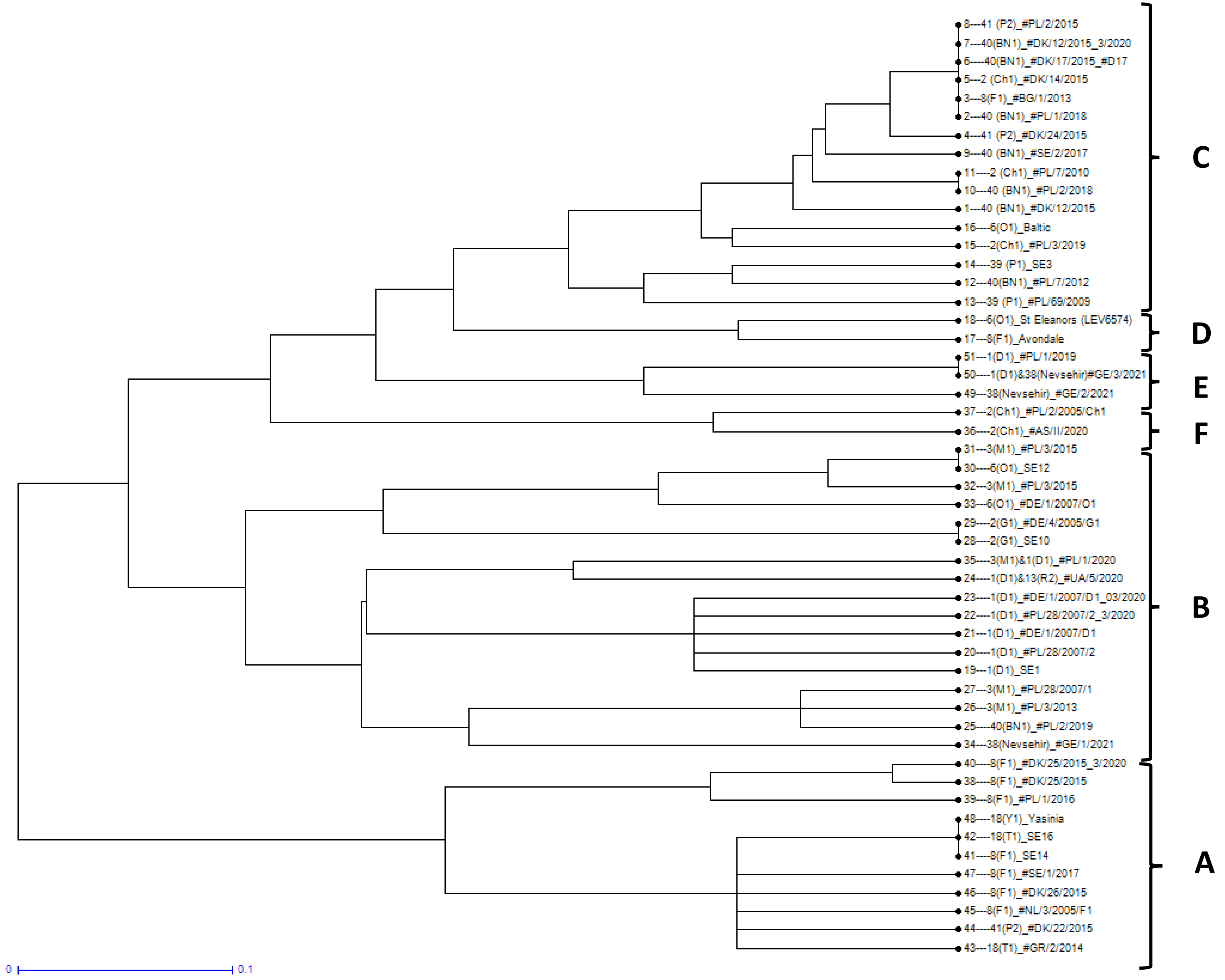

Figure S1e: UPGMA dendrogram based on the Socal & Sneath dissimilarity index for the 51 isolates using 73 marker-alleles.
